# A CKD Patient with Microcystic Kidneys on Imaging

**DOI:** 10.34067/KID.0000000000000200

**Published:** 2023-09-28

**Authors:** Bhavna Bhasin-Chhabra, Maitray D. Patel, Abhilash Koratala

**Affiliations:** 1 Division of Nephrology and Hypertension, Mayo Clinic, Scottsdale, Arizona; 2 Department of Radiology, Mayo Clinic, Phoenix, Arizona; 3 Division of Nephrology, Medical College of Wisconsin, Milwaukee, Wisconsin

**Keywords:** lithium, CKD, renal microcysts, renal imaging, ultrasound

## Abstract

**Figure:**
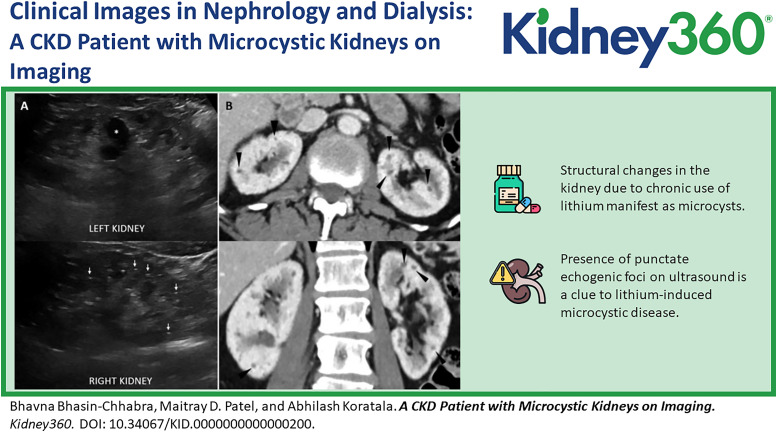

## Case Description

**Figure 1.**
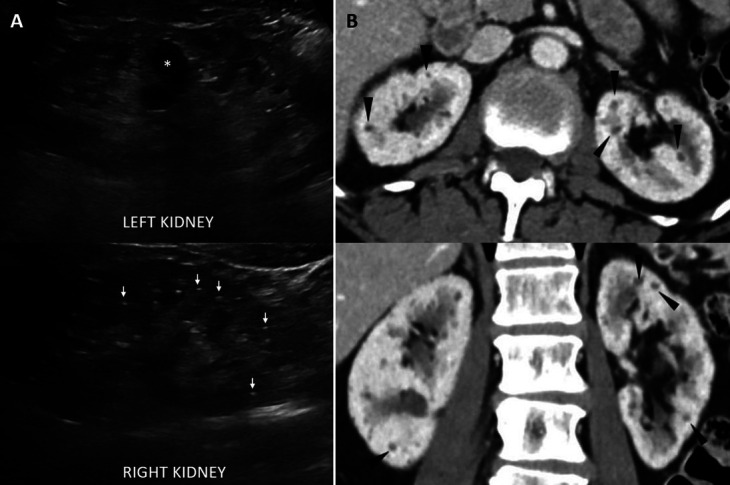
**Radiologic appearance of lithium induced kidney cysts.** (A) Kidney ultrasound images demonstrating multiple punctate echogenic foci and associated tiny echolucent areas (asterisk corresponds to a macrocyst and arrows point to a few representative microcysts). (B) Microcysts noted on CT imaging (arrowheads on a few representative lesions). CT, computed tomography.

A 68-year-old woman was seen in the nephrology clinic for evaluation of CKD. Her other medical problems included hypertension, osteoporosis, gastroesophageal reflux disease, hyperlipidemia, and bipolar disorder, which was treated with lithium (Li) for 10 years and now managed with valproic acid. Review of laboratory data revealed a relatively stable kidney function with a serum creatinine of 1.3 mg/dl. Urinalysis showed a specific gravity of 1.006 but was negative for any blood or protein. Serum sodium and bicarbonate levels were within normal limits. A kidney ultrasound was obtained to exclude structural abnormalities, which revealed numerous bilateral scattered punctate echogenic foci in the parenchyma suggestive of Li-induced microcysts (Figure 1A). In addition, a few macrocysts measuring up to 2 cm were found in both kidneys. The kidney length was normal (10.8 and 10.4 cm on the right and left, respectively). Review of a prior computed tomogram (computed tomography scan) confirmed the findings (Figure 1B). Because the patient was already off Li, she was managed conservatively with longitudinal monitoring of kidney function.

## Discussion

Li has been an essential drug for the management of bipolar disorder and has been in clinical use for several decades. Nephrotoxicity is a drug-limiting adverse effect of Li and may lead to the development of CKD, nephrogenic diabetes insipidus, distal renal tubular acidosis, proteinuria, and interstitial nephritis. Li-induced microcysts in the kidney arise from dilatation of distal tubules or collecting ducts and are usually 1–2 mm in size.^1^ Variable mechanisms have been reported for the development of microcysts. These may be a sequela of Li-induced chronic tubulointerstitial nephritis^1^ or may be related to low turnover of renal tubular epithelial cells because of antiapoptotic effects of Li, thus allowing for increased growth of tubular cells and formation of microcysts from invaginating tubular cells in the cortex and medulla.^2^ It has been postulated that an increase in size of the cysts over time may contribute to the development of CKD.^2^ Microcysts can also be visualized on computed tomography or magnetic resonance imaging, although punctate echogenic foci noted only on renal sonography may precede the appearance of changes on these modalities.^3^ The origin of these echogenic foci is interesting because cysts normally appear as anechoic (dark) structures on ultrasound. However, they appear echogenic (bright) in this scenario likely because of specular reflections from microcysts, which are smaller than the threshold of ultrasound resolution. Microcalcifications and colloid within the cysts are other potential causes of this echogenicity, but these findings were not consistently demonstrated in histopathologic studies.^3,4^ Of note, these kidney parenchymal changes may not be apparent when using low-resolution handheld point-of-care ultrasound devices.

## Teaching Points

- Structural changes in the kidney due to chronic use of lithium manifest as microcysts.
- Presence of punctate echogenic foci on ultrasound is a clue to lithium-induced microcystic disease.

## Disclosures

M.D. Patel reports the following: Honoraria: UpToDate. All remaining authors have nothing to disclose.
